# Evolution of Collective Behaviour in an Artificial World Using Linguistic Fuzzy Rule-Based Systems

**DOI:** 10.1371/journal.pone.0168876

**Published:** 2017-01-03

**Authors:** Jure Demšar, Iztok Lebar Bajec

**Affiliations:** Faculty of Computer and Information Science, Večna Pot 113, 1000 Ljubljana, Slovenia; Peking University, CHINA

## Abstract

Collective behaviour is a fascinating and easily observable phenomenon, attractive to a wide range of researchers. In biology, computational models have been extensively used to investigate various properties of collective behaviour, such as: transfer of information across the group, benefits of grouping (defence against predation, foraging), group decision-making process, and group behaviour types. The question ‘why,’ however remains largely unanswered. Here the interest goes into which pressures led to the evolution of such behaviour, and evolutionary computational models have already been used to test various biological hypotheses. Most of these models use genetic algorithms to tune the parameters of previously presented non-evolutionary models, but very few attempt to evolve collective behaviour from scratch. Of these last, the successful attempts display clumping or swarming behaviour. Empirical evidence suggests that in fish schools there exist three classes of behaviour; swarming, milling and polarized. In this paper we present a novel, artificial life-like evolutionary model, where individual agents are governed by linguistic fuzzy rule-based systems, which is capable of evolving all three classes of behaviour.

## Introduction

The intricate patterns of collective motion, observable in flocks of birds, schools of fish, herds of ungulates, swarms of insects, and human crowds [1–4] are a special treat. It is no wonder that the study of computational modelling of collective behaviour has a broad interdisciplinary appeal, more so as recent studies suggest similar patterns even in cancerous cells [5]. Researchers come from various areas: ethology, biology, mathematics, physics, computer science and robotics/control theory.

The first attempts at modelling collective behaviour date to the early 1980s, when Aoki [6] and Okubo [7] proposed an individual-based approach to the simulation of schooling mechanisms in fish, but it was Reynolds’ 1987 seminal paper [8] that attracted computer scientists to the field. Current models are presented by an interdisciplinary community and are either minimalistic with the goal of being as mathematically tractable as possible [9–14], or far more complex than the early ones [15–19]. However, the basic principles have stayed the same for over 30 years of active research.

Nowadays, collective behaviour is most often modelled on a per individual basis, where one models a single individual and observes the emergent behaviour that arises when a number of these individuals interact, thus closely following Aristotle’s concept of “the whole is more than the sum of its parts.” An individual is typically modelled as a multi-stage process [20, 21], in the minimal form consisting of perception, drives and action selection. Perception mimics the animal’s act of filtering out only the most important information about the surrounding environment (in most cases this is a subset of position and orientation data about nearby neighbours). Drives reproduce the modelled animal’s needs, where (with the notable exception of the most minimalistic models) these are typically the animal’s tendency to a) avoid collisions with nearby neighbours, termed separation, b) to surround itself with neighbours, termed cohesion, and c) to align in speed and velocity with neighbours, termed alignment. As the drives are potentially contradictory, e.g. separation and cohesion, the third stage, action selection, is responsible for devising the final action of the modelled animal, typically a change in heading and/or speed. Most of the models encode the drives by means of equations, where for example cohesion is typically encoded as a force vector directing the individual towards the centroid of nearby neighbours [2, 3]. Action selection is then most often a weighed sum of force vectors; actions that would fulfil individual drives.

A review of biological literature suggests that since the early days not much has changed in view of encoding the animal’s drives, whereas a lot of research has been devoted to perception and interaction. All because it is still not completely known ‘how’ when a flock of tens of thousands individuals is turning and wheeling it seems that all turn at once, reminiscent of ‘thought transference’ or ‘telepathy’ [3, 22]. Ballerini *et al*. [23] based on 3D data collected from live flocks of birds argued that interaction is not metric, i.e. distance limited, as in [8], but rather topological, i.e. number limited. While some earlier studies proposed a zone based interaction [15, 24] and later studies investigated the effects of visual occlusion [18, 25, 26] current research suggests that one can reproduce empirical data from physicists in Rome [23, 27] if either a) one assumes the probability of interaction between two individuals is inversely proportional to their distance [28], or b) one assumes that individual birds avoid a single closest neighbour only and they align with and are attracted to their seven closest neighbours [29], as assumed in Ballerini *et al*. [23].

The use of fuzzy logic as the means of encoding the simulated animal’s drives was first proposed in [30–33] and later followed by others [34–41]. While with the premise of easing the construction of new, yet unknown, drives to ethologists the first studies proposed a linguistic fuzzy rule-based system the later ones were more control centric and most of them proposed a Takagi-Sugeno rule base. A linguistic fuzzy rule-base was used also in [40] and [39], but contrary to [32] where a) the drives are encoded indirectly, b) no-uncertainty is assumed and c) collective behaviour emerges with no designated leader, [40] was based on the leader-follower concept and [39] while concentrating on noisy sensor measurements presented an interval-valued fuzzy controller.

Some researchers from the artificial life community might argue that most of the aforementioned models are in essence top-down models of collective behaviour in that their drives were designed by observing high-level behaviours of the group, regardless if these same drives dictate the behaviour of the individuals in the group. For a true bottom-up model then, where the individuals have their own individual movement rules that ‘may’ lead to collective behaviour when simulated, one would need to investigate the evolution of an individuals’ movement rules. Indeed, regardless of all research that has been done the biological question ‘why’ collective behaviour evolved still remains largely unanswered. Here the interest goes more into which pressures have led to the evolution of collective animal behaviour. From the early days of research in the field several studies attempted the evolution of collective behaviour. The methods range from genetic programming with LISP [42], PUSH [43], neuroevolution [44–46] to evolutionary optimization of the complete sensory-motor flow via Kuramoto oscillators bound to a synthetic optic flow retina [19]. These studies were mostly concerned if an evolutionary computation system can be used to evolve collective behaviour from scratch and had various degrees of success, where the latter can most often be attributed to steered evolution. More recently evolutionary models are being used to test the biological hypothesis that collective behaviour evolved due to combined search for food resources [47], or as protection from predation [48], which can be split further by the key ideas; selfish herd [49–54], predator confusion [55–59], many eyes [60, 61], and dilution of risk [62]. Most of the aforementioned evolutionary models use genetic algorithms to tune a) the importance of known drives (cohesion, separation, alignment) and/or additional drives (e.g. escape), and/or b) model parameters (field of view, escape distance, etc.). Very few studies attempted and successfully evolved collective behaviour from scratch, and in these cases the evolved behaviour can be termed as ‘crude’; portraying only clumping [47, 63], or swarming with collisions [47, 52, 53, 58].

In this paper we present an evolutionary model where the drives are encoded by means of linguistic fuzzy rule-based systems and is capable of evolving more ‘refined’ behaviours.

## Materials and Methods

Our model is an individual-based model consisting of predator and prey agents that coexist in a 2D environment (artificial world). The simulation runs at discrete updates, where each individual agent (predator or prey) based on the perceived state of the environment computes its drives, and with respect to the desired change in speed and heading updates its velocity and position [32, 33]. For the sake of simplicity the time steps and distances in the simulations are given in arbitrary units, have no physical meaning and are used for comparative purposes only. To keep the model’s complexity as low as possible we also assume constant, but different speeds for predator and prey agents. The following sections provide more details about the implementation of the predator and prey agents, as well as specifics about the evolutionary process.

### The predator agent

Since some avian visual sit-and-wait predators scan the neighbourhood by turning their head [64, 65] and some aquatic predators perform area-restricted search [66], our predator agents are capable of perceiving prey agents regardless of their relative bearing. For simplicity reasons, perception is limited only by distance, i.e. we also do not consider visual occlusion. Following our previous research [18] the predator agent’s drives for target prey pursuit are encoded by means of a linguistic fuzzy rule-based system and pre-set, i.e. excluded from evolution.

The goal of predator agents is to capture prey agents. We let multiple predator agents coexist, as recent studies suggest that the cumulative effect of high frequency attacks (through disorganisation of school cohesiveness) may increase the feeding success of each individual [67, 68]. Individual predator agents enter the artificial world after an initial random interval of update steps (*re-enter time*). Each predator agent continuously attacks prey agents for *hunt duration* update steps, then they are removed from the environment and a new predator agent enters after a random interval of update steps (*re-enter time*). Predator agents appear at random locations that are *ambush distance* from the artificial world centre, with their initial heading towards the centre. An attack involves target-selection, pursuit and capture attempt.

Following previous research [18, 53, 69] we implemented four target-selection tactics; attack the nearest prey agent, attack the most isolated prey agent, attack the most central prey agent, and high density area attack. Predators that attack the nearest, most isolated or most central prey individual, focus their attention on a single member of the prey group (ST), i.e. they select a single prey agent as target, and pursue it until captured. Predators that attack high density areas are larger than a single prey individual and do not select as target, pursue, and capture a single prey individual, but can capture several prey individuals in a single predation event. In view of recent results that attribute the evolution of clumping to the dilution of risk [63] rather than predator confusion [58], we opted to model our agents as non-confusable. This served also not to overly promote collective behaviour, as one could argue that outside attacks on the nearest or most isolated prey individual in combination with confusion pressures prey into grouping and thus might be viewed as a form of steered evolution. Additionally, if from an evolutionary perspective outside attacks on the nearest or most isolated prey individual have a positive influence on grouping, attacks on the most central prey individual or high density area attacks (HDA) should have a negative one [53]. Since our primary interest was the discovery of a wide variety of behaviours, we for this reason let the specific tactic an individual predator agent uses be chosen randomly (uniform probability). The complete set of predator agent parameters can be viewed in Table 1.

**Table 1:** Predator Agent Parameter Values.

Description	Value	
number of predator agents	16	
ambush distance	400	
re-enter time (update steps)	600–1200	
hunt duration (update steps)	600	
type	ST predator	HDA predator
size	6	12
speed	3	1.5
perception distance	500	500
catch distance	6	12

### The prey agent

Similar to predator agents, the prey agent’s perception is limited by distance only, i.e. we ignore the visual field blind area and visual occlusion. However, following recent research on perception models capable of reproducing empirical data [28, 29] we chose to model the probability of interaction between two prey agents as inversely proportional to their distance. Since every individual prey agent’s drives are encoded by means of a linguistic fuzzy rule-based system and evolve through time, the approach goes also in our favour as it reduces the number of rules that have to be evaluated per agent.

The goal of prey agents is to live as long as possible in the artificial world. When born, a prey agent is assigned a specific amount of *initial energy* (see Table 2) and on every update step it is rewarded for ‘living’ (*foraging gain*). Prey agents that collide or wander outside a square living area are penalized with a reduction of energy. One could argue that the *wandering penalty* is a form of steered evolution, but the *living area side length* was set to a large enough size that this does not overly promote grouping behaviour (see S1 Fig and S1 Video). The living area represents an area rich with food, shelter, or in another sense, attractive area. In our model the only purpose of this area is to keep prey agents inside a limited area of the artificial world, which could be achieved also by using periodic boundary conditions to create an infinite lattice, but we opted for an approach where prey agents learn to keep within the restricted area themselves.

**Table 2:** Prey Agent Parameter Values.

Description	Value
number of prey agents	100
spawn area radius	325
living area side length	375
size	1
speed	2
perception distance	100
initial energy	1000
foraging gain	1
collision penalty	-10
wandering penalty	-10

A prey agent dies when its energy drops to 0 or is caught by a predator agent. When a prey agent dies it is removed from the environment and a new prey agent is created, so that the group of ‘live’ prey agents is kept constant through time. The new prey agent, initially heading in a random direction, appears at a random location on a closed disc centred to the living area (*spawn area*). The linguistic rule base of the new prey agent is subject to evolution via crossover and mutation (see Evolutionary process).

**Fig 1:**
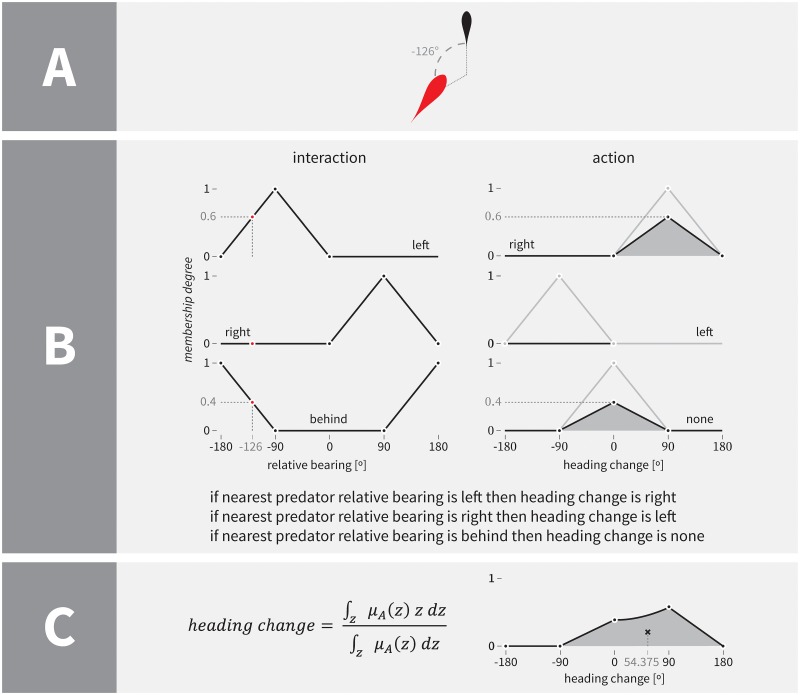
A simplified example of fuzzy reasoning. Section A shows the current state of the artificial world. The observed prey agent is depicted in black and the nearest predator in red. In this simplified example the observed prey agent performs fuzzy reasoning solely based on the nearest predator’s relative bearing (in this case -126°). The left part of section B presents the evaluation of the degree of truth of the antecedents of individual if-then rules that are listed at the bottom of this section. We assume no uncertainty in the data and model all inputs as singleton fuzzy values. For example, the degree of truth of the antecedent “nearest predator relative bearing is left” is therefore computed as 〈−180, −90,0〉 (−126) = 0.6. The right part of section B presents fuzzy inference or the evaluation of the consequent part of individual rules. Since we use the *product* t-norm (*x* ∘ *y* = *xy*) for implication this translates to scaling the triangular fuzzy number that is used to define the corresponding output linguistic variable’s value (shaded areas). For example, in the case of consequent “heading change is right,” this means 0.6 ∘ 〈0, 90, 180〉. Section C presents the aggregation of individual consequent parts and based on that the computation of the final, crisp output, the conclusion (desired change in heading). We aggregate rules via the *probabilistic sum* s-norm (*x* ◇ *y* = *x* + *y* − *xy*), and compute the crisp output by means of the *centre-of-gravity* defuzzification method. This means that the shaded area in section C (aggregated shaded areas from section B) gets translated into 54.375, the desired change in heading for the observed prey agent in this simplified example. For further details on fuzzy reasoning in general and its application to the modelling of collective behaviour consult [30–33, 70].

The prey agent is capable of perceiving a) the distance, relative bearing, and relative heading of the interacting prey individual, b) the distance, relative bearing, and relative heading of the nearest predator, as well as c) the distance and relative bearing to the closest point on the square that represents the outside edge of the living area. We assume no uncertainty in the data and model all inputs as singleton fuzzy values [70]. If either all other prey individuals, predators, or living area borders are outside of the prey agent’s perception distance, the corresponding linguistic variables are set to null, so that none of the fuzzy rules that contain the variable as part of the premise fire. In fuzzy reasoning (see Fig 1) we use the *product* t-norm for conjunction and implication, aggregate rules via the *probabilistic sum* s-norm, and compute the crisp output (desired change in heading) by means of the *centre-of-gravity* defuzzification method. The linguistic variables (Table 3) are decomposed into linguistic values, which are defined as either triangular fuzzy numbers

$$\begin{array}{c} \mu \left(x\right)=\langle l,m,r\rangle =\max\left(\min\left(\frac{x-l}{m-l},\frac{r-x}{r-m}\right),0\right), \end{array}$$ (1)

or periodic triangular fuzzy numbers

$$\begin{array}{c} {\mu \left(x\right)=\langle l,m,r\rangle }_{c}=\max\left(\min\left(\frac{(x-l)\;\mathrm{mod}\;c}{(m-l)\;\mathrm{mod}\;c},\frac{(r-x)\;\mathrm{mod}\;c}{(r-m)\;\mathrm{mod}\;c}\right),0\right). \end{array}$$ (2)

**Table 3:** Prey Agent Fuzzy Data Base.

	Linguistic variable	Linguistic value	Triangular fuzzy number
interaction	distance	next	〈0, 0, 10〉
		close	〈0, 15, 40〉
		near	〈20, 40, 60〉
		away	〈50, 60, 100〉
		far	〈60, 100, 100〉
	relative bearing	left	〈−180, −90, 0〉
		in front	〈−90, 0, 90〉
		right	〈0, 90, 180〉
		behind	〈90, 180, −90〉_360_
	relative heading	left	〈−180, −90, 0〉
		same	〈−90, 0, 90〉
		right	〈0, 90, 180〉
		opposite	〈90, 180, −90〉_360_
living area	distance	next	〈0, 0, 10〉
		close	〈0, 15, 40〉
		near	〈20, 40, 60〉
		away	〈50, 60, 100〉
		far	〈60, 100, 100〉
	relative bearing	left	〈−180, −90, 0〉
		in front	〈−90, 0, 90〉
		right	〈0, 90, 180〉
		behind	〈90, 180, −90〉_360_
action	heading change	hard left	〈−180, −180, −90〉
		left	〈−180, −90, 0〉
		none	〈−90, 0, 90〉
		right	〈0, 90, 180〉
		hard right	〈90, 180, 180〉

### Evolutionary process

Genetic algorithms have a long history in providing learning and adaptation capabilities to fuzzy rule-based systems [71, 72]. While in most applications the desired outcome of the evolutionary process is a better, faster, more accurate or interpretable rule-based system [73–75] in our case the goal of the evolutionary process is discovery through exploration. In other words the only objective considered by our fitness function is the survivability of prey agents, assessed via their energy level and therefore the fitness function does not consider collective behaviour directly.

In our artificial world predator and prey agents coexist and the goal of predator agents is to capture prey agents, while the goal of prey agents is to survive. The direct competition of individual prey agents by way of their drives (i.e. rules of motion encoded via a linguistic fuzzy rule-based system) will lead to the emergence of collective behaviour only if such behaviour helps individual prey agents to ‘live’ longer. Artificial life based evolutionary computation like this tries to mimic open-ended evolution [76–78]. To our knowledge there have been only few similar evolutionary fuzzy systems [79, 80] and none devoted to the evolution of collective behaviour.

As we wished to focus on human interpretable rules and keep the complexity as low as possible, we opted to use a fixed data base and evolve only the rule base [81]. In addition, as the order of importance of individual inputs is unknown, we allowed for incomplete rule sets, i.e. we limited only the number of rules in the rule base as well as the number of antecedents per individual rule.

To recapitulate, prey agent behaviour was evolved via an open-ended like evolution where the behaviour of an individual prey agent is defined by the complete fuzzy rule base with a variable-length (messy) coding scheme [82]. The chromosome of each individual was thus its set of rules, in genetic fuzzy systems labeled as the Pittsburg approach [71]. Individual prey agents of the initial population were assigned random behaviours (i.e. a set of random fuzzy rules). They were placed at random locations on a closed disc centred to the living area and assigned random headings. When a prey agent was caught by a predator agent or died due to numerous collisions or wanderings outside of the living area, it was removed and a new prey agent was created. Two ‘live’ prey agents were chosen as its parents, where selection was fitness-proportional. The fuzzy rule base of the new prey agent was constructed by first choosing a random rule base length, and then randomly selecting individual rules from the joint sets of its parents’ rules. Following that, a mutation could occur; it triggered either an addition of new totally random rules or removal of existing rules from the new prey agent’s rule base.

## Results and discussion

We performed 20 individual evolutionary runs (Table 4). Each evolved behaviour was then evaluated by running 20 replicates of a separate simulation (Table 5). In this simulation, prey agents were initially placed at random locations on a closed disc centred to the living area and assigned random headings. After an initial stabilisation period, the observed parameters were first recorded without the presence of predators; only then a predator was introduced and another set of observed parameters was recorded.

**Table 4:** Evolutionary Process Parameter Values.

Description	Value
number of evolutions	20
total length (update steps)	10000000
rule base upper bound	50
antecedents upper bound	4
mutation probability	2%
upper bound of add rules mutation	3
upper bound of remove rules mutation	3

**Table 5:** Validation Process Parameter Values.

Description	Value
replicates of validation	20
stabilisation period (update steps)	900
predator introduction (update step)	1800
total length (update steps)	3600

### Behaviour analysis

**Fig 2:**
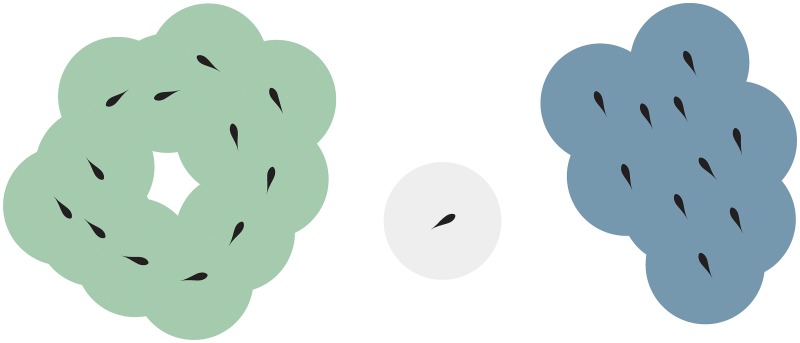
Prey agents that can influence each other either directly or indirectly via others are considered as being part of the same group. Presented are two groups (one in a milling state, green shading, and one in a polar state, blue shading) and one straggler (a prey agent that can neither influence nor be influenced by any other prey individual, grey shading).

For the analysis of the evolved behaviour we resorted to both visual inspection [56] and biologically relevant observables. Here we concentrated on local density [83], number of groups [20, 84], polarization and rotation [2, 15, 85]. Polarization

$$\begin{array}{c} p=\frac{1}{n}\left|\sum_{i=1}^{n}{\hat{v}}_{i}\right|, \end{array}$$ (3)

where *n* is the number of agents and ${\hat{v}}_{i}$ is the unit direction vector of agent *i*, provides a measure of how aligned the individuals in a group are. Rotation

$$\begin{array}{c} m=\frac{1}{n}\left|\sum_{i=1}^{n}{\hat{c}}_{i}\times {\hat{v}}_{i}\right|, \end{array}$$ (4)

where ${\hat{c}}_{i}$ is the position of agent *i* in the local coordinate frame of the group, on the other hand, expresses the degree of rotation of the group about its centre. Polarization and rotation were computed on the local scale, i.e. considering only prey agents that are part of the same group, as well as on the global scale, i.e. considering all prey agents as being part of one single group. Groups were established based on potential interaction (direct or indirect) [20, 84]. In other words, for an observed prey agent, all prey individuals that were inside its perception distance were considered as being part of its group, as well as, recursively all prey individuals that were inside the perception distance of any of this group’s members. Prey agents that had all prey individuals outside their perception distance were marked as stragglers and excluded from the analysis on the local scale (see Fig 2).

As based on the relation between polarization and rotation Couzin *et al*. [15] defined four classes of collective behaviour, namely *swarming*, *milling*, *dynamic parallel group* and *highly parallel group* we, in addition to assessing the behaviour visually, also classify it based on the corresponding representative values of polarization and rotation. Here we followed recent research by Tunstrøm *et al*. [85], who defined that a group is in: the polar state (P) when polarization > 0.65 and rotation < 0.35; the milling state (M) when polarization < 0.35 and rotation > 0.65; and the swarm state (S) when polarization < 0.35 and rotation < 0.35. Outside these ranges it is said to be in transition (T).

**Fig 3:**
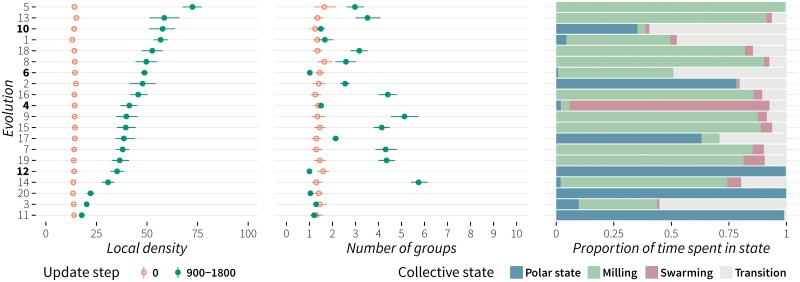
Local density, number of groups, and time spent in a specific collective state for each evolutionary run. The left graph displays the mean and bootstrapped 95% CI of the local density at update step 0, as well as, mean and bootstrap 95% CI of the average local density during update steps 900–1800. The middle graph displays the mean and bootstrapped 95% CI of the number of groups at update step 0 and the mean and bootstrapped 95% CI of the average number of groups during update steps 900–1800. The right graph shows the cumulative proportion of time spent in a specific collective state. The number of replicates was 20. Evolution ids were sorted based on the average local density during update steps 900–1800. Ids marked in bold correspond to representatives of the four classes of evolved behaviour (see main text for details).

As it can be seen in Fig 3, all 20 evolutions led to an increase in local density. Overall the mean local density at update step 0 was 14.07 (95% CI, 13.87–14.26), and the average local density during update steps 900–1800 was 42.47 (95% CI, 40.91–44.1). It ranged from 17.74 (95% CI, 16.46–19.18) in the case of evolution no. 11, to 72.47 (95% CI, 67.85–77.36) in the case of evolution no. 5. In all cases the increase was statistically significant (*p* < 0.0001). The overall average number of groups during update steps 900-1800 was 2.762 (95% CI, 2.603–2.932), and ranged from 1.001 (95% CI, 1–1.002) in the case of evolution no. 12 to 5.743 (95% CI, 5.374–6.122) in the case of evolution no. 14.

**Fig 4:**
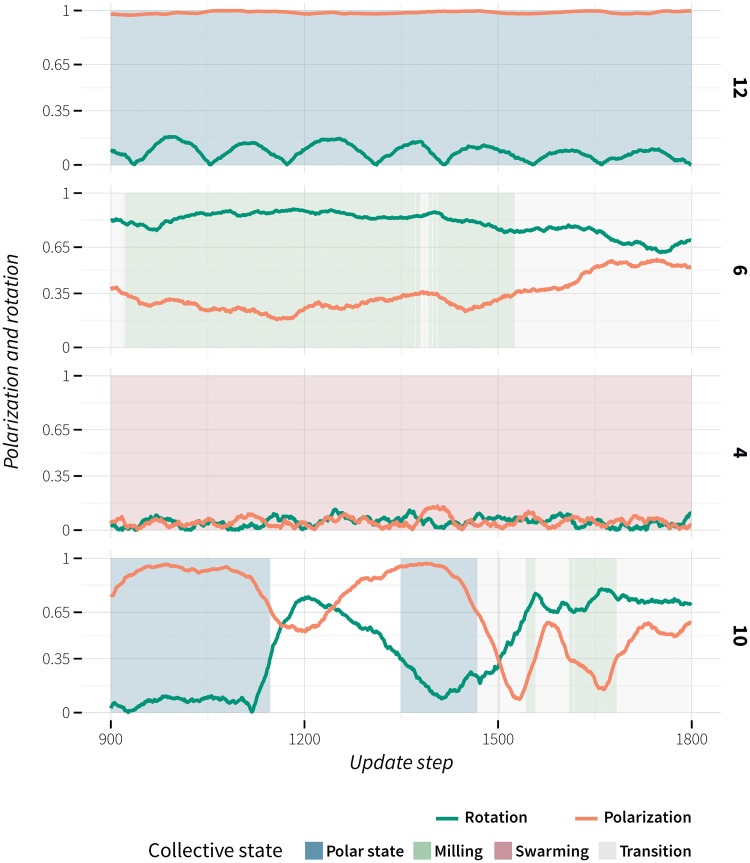
Time series of global polarization and rotation for each of the four classes of evolved behaviour. Evolutions no. 12, 6, 4 and 10 (marked as bold in Fig 3) were selected as representatives of polarized, milling, swarming and dynamic behaviour based on the proportion of time spent in a specific state, high mean local density and low mean number of groups. For video sequences of the representative evolved behaviours see S2–S6 Videos.

The proportion of time spent in a specific collective state (P, M, S, T) was determined by counting the cumulative number of update steps over all 20 replicates that individual groups spent in a specific state. When there was more than one group, each group’s state was allocated a corresponding proportion of update steps, e.g. when there were three groups in one step their respective states were assigned one third of the update step each. Based on the state in which the largest proportion of time was spent in, the evolved behaviours were classified as: polarized (evolutions no. 2, 11, 12, 17 and 20), milling (evolutions no. 5–9, 13–16, 18, and 19), and swarming (evolution no. 4). Evolutions no. 1, 3 and 10 spent the largest proportion of time in transition between states. This was confirmed through visual inspection. As we also noticed that the groups continuously transitioned between different states (polarized-milling-swarming), a characteristic associated with schooling fish (golden shiner, *Notemigonus crysoleucas*) [85], we classified this type of evolved behaviour as *dynamic* (D). Fig 4 shows representative time series of global polarization and rotation for each of the four types of evolved behaviour. Evolutions no. 12, 6, 4 and 10 were selected based on high mean local density and low mean number of groups (marked as bold in Fig 3).

**Fig 5:**
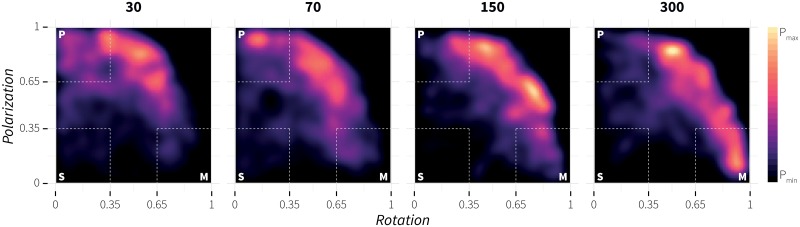
Density plot of global polarization versus rotation for various group sizes in the case of evolution no. 10. The density plots visualize the relationship between group size and behaviour stability. Increasing the number of agents leads global behaviour to change from predominantly polarized to predominantly milling.

To gain further understanding of the evolved behaviour in the case of evolution no. 10, we performed an experiment similar to the one Tunstrøm *et al*. [85] used to evaluate the relationship between group size and behaviour stability. Note that a) in our case the speed was not varied, but kept constant (see Table 2), b) in our case there was a boundary interaction (living area), c) global polarization and rotation were recorded during update steps 900–1800, e) 20 replicates were performed, and d) the rule bases of the 30, 70, 150, 300 agents were on each replicate chosen randomly from the pool of 100 rule bases that resulted from evolution no. 10. The density plot in Fig 5 shows qualitatively similar results to those presented by Tunstrøm *et al*. [85]. With increasing the number of agents, the global behaviour changes from predominately polarized to predominately milling. In addition, visual inspection revealed that, like in the case of schooling fish [85], transitions from polarized to the milling state and back were initiated mainly by a) interaction with the living area boundary or b) agents located in the frontal region of the group, which after a turn spotted the back of the group (see S5 Video).

A Wilcoxon Signed-Ranks Test was used to compare the local density and number of groups during update steps 1800–3600, in which the predator was not present, to the local density and number of groups during these update steps in which the predator was present. With the exception of evolution no. 3, the presence of a predator caused a statistically significant (*p* < 0.01) change in local prey density and the number of groups. An increase in local density in combination with a decrease in the number of groups was seen in evolutions no. 1, 10 and 11. A decrease in local density in combination with an increase in number of groups was seen in evolutions no. 2, 4, 5, 7, 9, 12, 13, 16, 17, 19. A decrease in both local density and number of groups was noted in evolutions no. 6, 14, 15, 18 and 20. Finally, an increase in both local density and number of groups occurred in the case of evolution no. 8. In the case of evolution no. 3 no significant difference was observed in both local density (*Z* = −0.786, *p* = 0.432) and the number of groups (*Z* = −1.645, *p* = 0.1). Visual inspection revealed that even in the case of evolution no. 3 individual prey agents did react to predators by turning away from it, however this had no notable effect on local density or the number of groups.

### Rule base analysis

Since in our case the data base was fixed, the evolved rule bases were summarized by means of six parameters, namely *living area*, *predator*, *prey*, *specificity*, *bias*, and *size*. The first three were computed as the average proportion of rule antecedents that contain linguistic variables related to the living area, nearest predator, and interacting individual, respectively:

$$\begin{array}{c} \rho^{\alpha }=\frac{1}{n}\sum_{i=1}^{n}\frac{a_{i}^{\alpha }}{a_{i}}, \end{array}$$ (5)

where *n* is the number of rules in the rule base, *a*_*i*_ the number of antecedents in rule *i*, *α* either living area, nearest predator, or interacting individual, and $a_{i}^{\alpha }$ the number of antecedents in rule *i* that contain linguistic variables related to *α*. These three parameters provide a rough approximation of the amount of attention the prey agent gives to a specific aspect of the artificial world. The parameters sum to 1. Since the living area is described with two linguistic variables, and the nearest predator and the interacting individual are with three, an equal attention to all three aspects would result in their values being 2/8, and 3/8, respectively.

Rule base specificity was determined as:

$$\begin{array}{c} \varsigma =\frac{1}{n}\sum_{i=1}^{n}\frac{a_{i}-1}{m-1}, \end{array}$$ (6)

where *n* is the number of rules, *m* the maximum number of antecedents (antecedents upper bound in Table 4), and *a*_*i*_ the number of antecedents in rule *i*. A value of 0 indicates all rules in the rule base use only one antecedent, thus individual rules are very general as the outcome is determined by a single input only. A value of 1, on the other hand, indicates that all rules in the rule base use the maximum number of antecedents, thus individual rules are highly specific as the outcome is determined by the highest number of inputs.

Bias provides a rough approximation of the prey agent turning side preference. It was computed as:

$$\begin{array}{c} \beta =\frac{1}{2n}\sum_{i=1}^{n}1+\frac{o_{i}}{180}, \end{array}$$ (7)

where *n* is the number of rules, and *o*_*i*_ is the centroid of the output linguistic value of rule *i*. Values below 0.5 thus indicate a bias towards left turns, and values above 0.5 a bias towards right turns. Last but not least, size is simply the number of rules in the rule base divided by the maximum number of rules possible (rule base upper bound in Table 4).

We first noted that overall prey agents based their decisions more on predator related linguistic variables (Mdn = 0.385) than interacting individual related ones (Mdn = 0.361; *Z* = −19.591, *p* < 0.0001). The amount of attention given to the living area (Mdn = 0.255) was significantly different (*Z* = −3.885, *p* = 1.025e-4) than what would be expected if an equal attention was given to all three aspects of the artificial world. Similarly, a slight preference for turning to the right is present (Mdn = 0.507; *Z* = −8.796, *p* < 0.0001).

**Fig 6:**
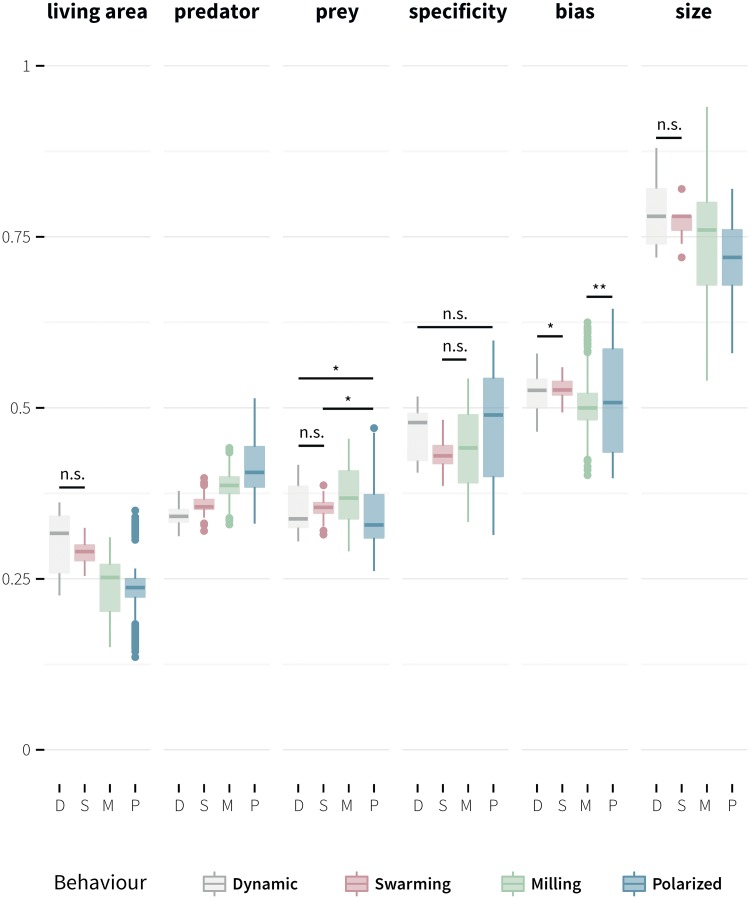
Box plots of the distributions of the six parameters through which the evolved rule sets were summarized. Grouping was based on the evolved behaviour class. Statistical significance of differences was obtained by means of a Benjamini-Yekutieli adjusted Dunn test. Symbols ns, * and ** indicate *p* ≥ 0.05, *p* < 0.05, and *p* < 0.01, respectively. Unmarked cases denote significance at *p* < 0.001.

The evolved rule bases of individual evolutions were then grouped based on the type of evolved behaviour (polarized, milling, swarming, or dynamic) and a pairwise multiple comparison was made using a Benjamini-Yekutieli adjusted Dunn test. Fig 6 shows box plots of the distributions of all six parameters, and statistical significance of the inter group differences.

In the pairwise comparison between all four groups a difference at *p* < 0.001 was observed only in the case of the average proportion of rule antecedents that contain predator related linguistic variables. Interestingly non significant differences were observed between the dynamic and swarming group in the cases of a) the average proportion of rule antecedents that contain living area related linguistic variables, b) the average proportion of rule antecedents that contain linguistic variables related to the interacting individual, and c) rule base size. In the case of rule base specificity no statistically significant difference was observed between a) the dynamic and polarized group, and b) the swarming and milling group. The dynamic and swarming group had a significantly higher bias (Mdn = 0.526) than the polarized (Mdn = 0.508) and milling group (Mdn = 0.5). Surprisingly, in the case of the milling group, the median rank was not statistically different than 0.5 (*Z* = −1.919, *p* = 0.055), which indicates no preference for the side of turning.

## Conclusion

The study of collective behaviour has a broad interdisciplinary appeal. Numerous studies have attempted to evolve collective behaviour, most by tuning parameters of previously presented non-evolutionary models. Very few succeeded to evolve it from scratch, and even in these cases the evolved behaviour can be termed as ‘crude’. Based on presented images and available video footage they portray only clumping [47, 63], or swarming with collisions [47, 52, 53, 58]. In this work we have presented an open-ended, artificial life-like evolutionary model where the drives of individual agents are encoded via linguistic fuzzy rule-based systems. We analysed the evolved behaviour and showed that based on biologically relevant observables [2, 15, 85] the system is capable of evolving a wide range of behaviours, some qualitatively similar to those reported in experimental research [85]. Through the analysis of the evolved rule bases we have also shown that when grouping the evolved rule bases by the type of evolved behaviour and observing the average proportion of rule antecedents that contain predator related linguistic variables there exists a statistically significant difference between the evolved rule bases. We believe that artificial life-like evolutionary modelling based on linguistic fuzzy rule-based systems might prove very useful in answering the biological question ‘why’ collective behaviour evolved, and due to their linguistic nature also provide a deeper insight into the ‘how’.

## Supporting Information

S1 Fig Local density, number of groups and time spent in a specific collective state for control evolutionary runs. To verify that the living area does not overly promote grouping behaviour we performed 20 evolutionary runs with no predators present. Each evolved behaviour was evaluated by running 20 replicates of a separate simulation. For ease of comparison the graphs present the same data as in Fig 3, this time however for evolutionary runs with no predators present. In all cases there was practically no grouping. Note that local density in all but two cases decreased rather then increased. The prey agents learned to spread out over the entire living area, to stay inside the borders of the living area, and to avoid each other (prevent collisions). In all cases the local density, polarization and momentum were very low, which resulted in a scattered swarming behaviour (see S1 Video). Note that the evolved swarming behaviour is very different with respect to the behaviour that evolved in the case when predators were present (see S2 Video). In the latter case prey learned also to group and react to predator attacks.
(EPS)

S1 Video Video sequence portraying a representative behaviour for the case of evolutionary runs with no predator present. Local density, polarization and momentum are very low throughout the entire simulation run and the evolved behaviour can be classified as scattered swarming behaviour. The prey agents learned to spread out over the entire living area, to stay inside the borders of the living area, and to avoid each other (prevent collisions). Note that the evolved swarming behaviour is very different with respect to the behaviour that evolved in the case when predators were present (see S2 Video). In the latter case prey learned also to group and react to predator attacks.
(MP4)

S2 Video Video sequence portraying a representative of the evolved swarming behaviour (evolution no. 4). Polarization and momentum are very low throughout the entire simulation run and the evolved behaviour can be classified as swarming behaviour. Note that the local density is higher than in the case of evolutions with no predator present (see S1 Video). Prey agents learned to stay inside the borders of the living area, and to avoid each other (prevent collisions). They learned also to group (by circling each other in an unordered fashion) and react to predator attacks (see frames 3700–3800 and 4600–5000). Note that soon after the disturbances induced by the predator attacks the swarming behaviour re-stabilizes.
(MP4)

S3 Video Video sequence portraying a representative of the evolved milling behaviour (evolution no. 6). Polarization is low and momentum high throughout the entire simulation run and the evolved behaviour can be classified as milling behaviour. Note that the local density is higher than in the case when prey behaviour evolved with no predator present (see S1 Video). Prey agents learned to stay inside the borders of the living area, to avoid each other (prevent collisions) and group (by circling around an empty core in an ordered fashion), as well as react to predator attacks (see frames 1900–2100, 2700–2900 and 4700–5000). At frame 4900–4950 one can also observe the formation of a vacuole. Note that soon after the disturbances induced by the predator attacks the milling behaviour re-stabilizes.
(MP4)

S4 Video Video sequence portraying a representative of the evolved polarized behaviour (evolution no. 12). Polarization is very high and momentum low throughout the entire simulation run and the evolved behaviour can be classified as polarized behaviour. Note that the local density is higher than in the case when prey behaviour evolved with no predator present (see S1 Video). Prey agents learned to stay inside the borders of the living area, to avoid each other (prevent collisions) and group (by matching each other’s heading). Prey agents learned also to react to predator attacks (see frames 1800–2000, 2700–2900, 3600–3900 and 4500–5100). Note that soon after the disturbances induced by the predator attacks the polarized behaviour re-stabilizes. Note also that apart from one individual all other prey agents resort to grouping and polarized behaviour, whereas the aforementioned individual does so only occasionally, evidence that in our case the behaviours of prey agents are heterogeneous (see frames 900–2700). For this reason the individual, however, becomes an easy target for the predator that attacks peripheral prey (see frames 2700–2900).
(MP4)

S5 Video Video sequence portraying a representative of the evolved dynamic behaviour (evolution no. 10). Prey agents learned to stay inside the borders of the living area, to avoid each other (prevent collisions), to react to predator attacks (see frames 1900–2100, 2700–2900, 3700–3800 and 4700-5100), and to group. Note, however, that in contrast to S2–S4 Videos the prey agents in this case continuously transition between different states (polarized-milling-swarming) which results in the largest proportion of time spent in transition between states. This dynamic behaviour re-stabilizes soon after the disturbances induced by the predator attacks.
(MP4)

S6 Video HD video sequence where representative evolved behaviours can be observed simultaneously. A 2x2 HD version where video sequences from S2–S5 Videos are played in synchrony and can be observed simultaneously was constructed to ease the comparison of the representative evolved behaviours. Here available for download only. An online viewable version is available at https://vimeo.com/190425371. See captions of S2–S5 Videos for details.
(MP4)
